# Accidental radioisotope burns - Management of late sequelae

**DOI:** 10.4103/0970-0358.70725

**Published:** 2010-09

**Authors:** Bipin T. Varghese, Shaji Thomas, Balakrishnan Nair, P. C. Mathew, Paul Sebastian

**Affiliations:** Division of Surgical Oncology, Regional Cancer Centre, Trivandrum, India

**Keywords:** Non-healing ulcer, radioisotope burns, reconstructive surgery, unstable scar

## Abstract

Accidental radioisotope burns are rare. The major components of radiation injury are burns, interstitial pneumonitis, acute bone marrow suppression, acute renal failure and adult respiratory distress syndrome. Radiation burns, though localized in distribution, have systemic effects, and can be extremely difficult to heal, even after multiple surgeries. In a 25 year old male who sustained such trauma by accidental industrial exposure to Iridium192 the early presentation involved recurrent haematemesis, pancytopenia and bone marrow suppression. After three weeks he developed burns in contact areas in the left hand, left side of the chest, abdomen and right inguinal region. All except the inguinal wound healed spontaneously but the former became a non-healing ulcer. Pancytopenia and bone marrow depression followed. He was treated with morphine and NSAIDs, epidural buprinorphine and bupivicaine for pain relief, steroids, antibiotics followed by wound excision and reconstruction with tensor fascia lata(TFL) flap. Patient had breakdown of abdominal scar later and it was excised with 0.5 cm margins up to the underlying muscle and the wound was covered by a latissimis dorsi flap. Further scar break down and recurrent ulcers occurred at different sites including left wrist, left thumb and right heel in the next two years which needed multiple surgical interventions.

## INTRODUCTION

Accidental radioisotope burns is a rare occurrence. We present the case of a patient who suffered an accidental industrial exposure to Iridium^192^, the late sequelae of which was managed at our institution. The therapeutic interventions included epidural analgesia and multiple surgical excisions of unstable scars and reconstruction with flaps.

## CASE REPORT

**Figure 1:**
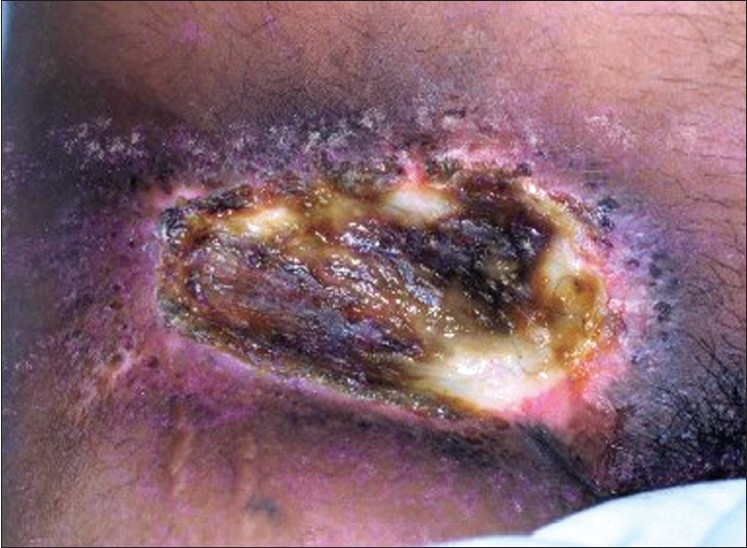
Ulcer measuring 5 × 5 cm in the right groin

A 25-year-old male, who sustained accidental industrial exposure to Iridium^192^ while repairing a gamma camera at his work place in Bahrain in March 2000, presented with a non-healing painful ulcer of size 5×5 cm over the right groin to our centre in October 2000 [Figure 1]. Further enquiry revealed that, immediately after exposure he developed recurrent attacks of hemetemesis requiring supportive treatment in a local hospital. Three weeks after the exposure he developed burns at the contact areas, which included the left hand, left side of the chest, abdomen and right inguinal region. All except the inguinal burn healed with unstable scars. At the fourth week he developed severe bone marrow depression with a platelet count of 61 × 10^9^/liters and a WBC count of 2.5 × 10^9^/liters, which also recovered with supportive treatment at the local hospital.

**Figure 2:**
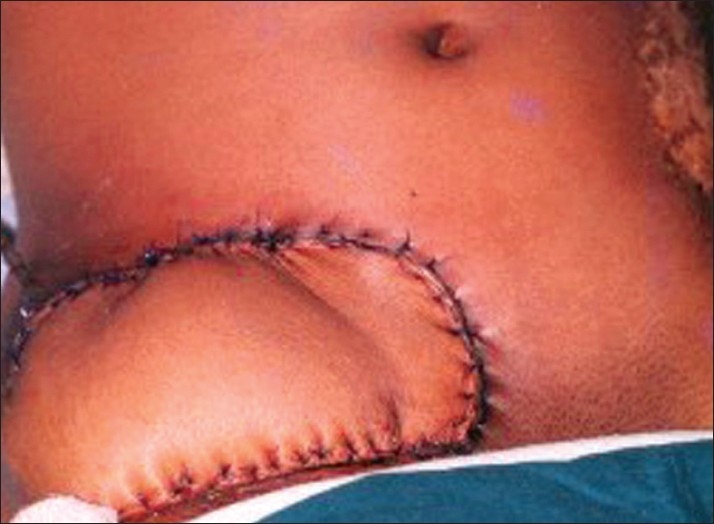
Reconstruction using TFL flap- immediate post operative view

**Figure 3:**
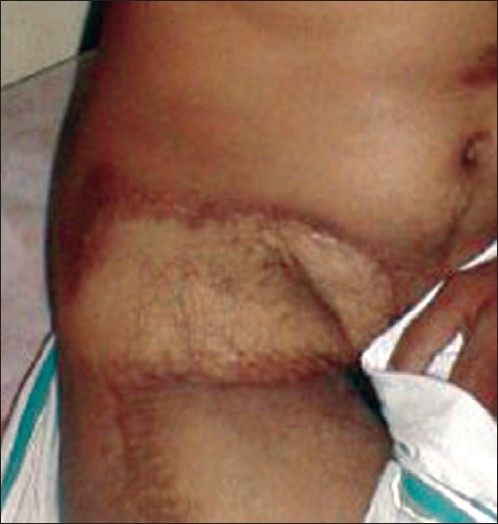
Reconstruction using TFL flap- late post operative view

The patient was admitted at the pain and palliative care ward and started on morphine and adjuvants, which included NSAIDs, steroids, antibiotics, and antidepressants after routine investigations were found to be normal. Due to inadequacy of pain relief with increasing doses of oral and intravenous morphine an epidural catheter was inserted through the second lumbar intervertebral space to deliver morphine buprinorphine and bupivacaine for round the clock analgesia. Once adequate analgesia was attained, the ulcer I the groin was excised with a margin of 0.5 cm all around, except at the depth where the femoral neurovascular bundle was situated, and covered by the adjoining groin flap under general anesthesia. An ipsilateral Tensor Fascia Latae (TFL) myocutaneous flap was used for cover of the groin flap donor site and the post operative period was uneventful [Figure 2]. The epidural catheter was removed on the fourth day post-operatively, and he was discharged on the 14^th^ day with NSAIDs for pain management [Figure 3]. However he was warned of further scar breakdown and hence advised regular follow up.

On 4^th^ April, 2001 he presented with another painful ulcer, 4 × 4 cm with surrounding unstable scar over the left side of the anterior abdominal wall. The groin flap had settled very well. The abdominal scar and ulcer were again excised with 0.5 cm margins up to the underlying muscle. An ipsilateral Latissimus Dorsi (LD) myocutaneous flap supplemented by a random transposition flap of size 6 × 4 cm was used to reconstruct the defect created by the excision. The LD donor site was closed primarily. Pain was managed by a protocol similar to that followed earlier, and he was discharged after an uneventful postoperative period with advice on further follow-up every two months.

**Figure 4:**
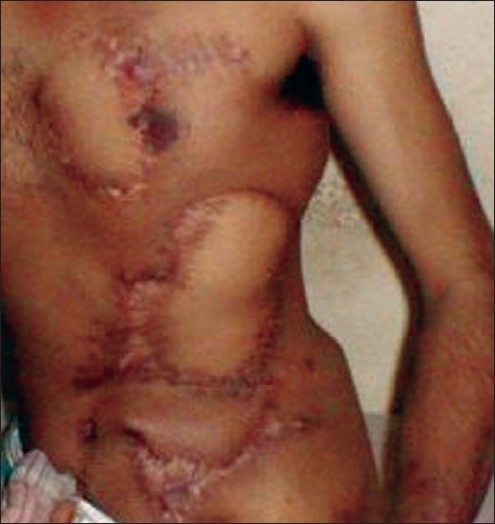
Late post-operative view of anterior abdomen and chest wall reconstruction

On 17^th^ October 2001 he again presented with a 3 × 3 cm ulcer with surrounding scar over the ulnar aspect of the left wrist. This time it was decided to prophylactically excise the unstable scar on the chest in addition to the ulcer on the wrist. The ulnar nerve was intact. Both defects were reconstructed with local transposition flaps. A fasciocutaneous transposition flap based on radial forearm perforators was used to close the wrist defect. The post-operative period was uneventful. There were no further episodes of scar breakdown and the patient returned to his job in Bahrain. On 2^nd^ March 2002, he presented with an ulcer on the volar aspect of the thumb tip for which a reconstruction with a Littler’s flap was advised. However, he did not report for surgery and on July 2002, presented with a fresh ulcer on the lateral aspect of the right heel. The thumb tip ulcer had almost healed with a healthy scar. After three months of pain and conservative management, the leg defect also started showing signs of healing. He is now on further follow up [Figure 4].

## DISCUSSION

Accidental exposure to industrial radiation is an occupational hazard the incidence of which is extremely rare due to the stringent safety measures at work places. Reports of accidental and significant individual exposure are sporadic and isolated.[1] The major components of radiation injury are burns, interstitial pneumonitis, acute bone marrow suppression, acute renal failure and adult respiratory distress syndrome.[2] On a cellular level, the damage caused by radiation can be either direct or indirect. Direct damage results from the “hits” or radiation absorption by the cells whereas indirect damage occurs when the radiation causes cellular water to release free radicals, which in turn combine to make cytotoxic peroxides. Direct effects of radiation can affect cellular DNA, which when altered may lead to cell destruction or aberrant cell replication and malignancy.[3] Although the pathological effects of radiation to the skin are well known, it is often difficult to assess the level of severity immediately and accurately, because of the delay between exposure and appearance of the lesions and because of hidden lesions in the underlying tissues. The severity of injury essentially depends upon the nature of the radiation, high energy penetrating radiation causing much more irreversible damage than low energy lightly penetrating radiation. Therefore, besides the clinical observation, diagnosis and prognosis should be based on several parameters such as dosimetry, reconstruction of the accident, thermography, and scintigraphy.[4]

Literature describes two types of “severe accidental radiation”: high dose localized radiation and accidental whole body overexposure. These two entities may coexist and may be associated with external or internal radioactive contamination. High dose localized irradiation is characterized predominantly by deep burns of the skin whereas in total body overexposure, hematological problems predominate.[5]

The acute management of severe accidental radiation includes resuscitation, supportive care, wound debridement and measures to reduce internal absorption.[1,3,6] The role of bone marrow transplant is very limited.[2] Overall management of radiation burns and its late sequelae is usually prolonged and challenging as in the present case.

Radioisotope burns are usually deep with a tendency for unstable scars. This is because of the vasculitis that ensues and the consequent decrease in blood flow to the deeper aspect of skin and subcutaneous tissues, and cellular damage to fibroblasts and their consequent depletion may also play a major role.[2,7] Pain is the most difficult medical problem to solve because it starts quickly, remains constant at all stages and dominates the clinical picture. Surgical treatment deals with deep ulceration and necrosis which is commensurate with the nature and energy of the radiation, the localization of the injury and its severity. The mainstays of surgical treatment are excision and grafting and/or reconstruction with flaps.[5] The most favorable time for intervention is difficult to specify, and should be neither too early before the establishment of the clinical picture nor too late.[4] When hand or flexural creases are involved aggressive debridement and immediate coverage with flaps is indicated.[8]
